# Sex differences in kidney and lung status in an animal model of brain death

**DOI:** 10.1016/j.clinsp.2025.100623

**Published:** 2025-03-26

**Authors:** Marina Vidal-dos-Santos, Roberto Armstrong-Jr, Maryna van Zil, Fernanda Yamamoto Ricardo-da-Silva, Lucas Ferreira da Anunciação, Mayara Munhoz de Assis Ramos, Cristiano de Jesus Correia, Petra J. Ottens, Luiz Felipe Pinho Moreira, Henri G.D. Leuvenink, Ana Cristina Breithaupt-Faloppa

**Affiliations:** a Laboratorio de Cirurgia Cardiovascular e Fisiopatologia da Circulação (LIM-11), Instituto do Coração (InCor), Faculdade de Medicina da Universidade de São Paulo, São Paulo, SP, Brazil; b Department of Surgery, University Medical Centre Groningen, University of Groningen, The Netherlands

**Keywords:** Sex differences, Rats, Brain death, Lung, Kidney

## Abstract

**Background:**

In transplantation, sex-mismatched procedures correlate with poorer outcomes. Previous research has indicated that females respond worst to Brain Death (BD) and that organ damage varies with the speed of BD induction. The authors aimed to investigate the effects of slow BD-induction on lung and kidney responses in male and female rats.

**Methods:**

Males and female rats were subjected to slow induction of BD and kept for 4h. Blood gas samples were taken at 0 h and 4h. At 4 h, blood, urine and tissue samples were collected. IL-1β was measured in plasma, lung homogenate and lung culture. IL-6 was quantified in plasma, lung culture, and kidney homogenate. Leukocyte infiltration/activation was evaluated. Biochemical analyses of creatinine were performed in the plasma. Naïve animals were used as controls.

**Results:**

Males presented reduced testosterone levels after 4h. Females presented reduced progesterone, whereas estradiol remained similar at 0 h and 4h. Compared with Naive, BD-groups presented increased plasma IL-1β and IL-6. Males and females presented reduced pO_2_ after BD, with females presenting even lower values at 4h. In lung tissue, males presented increased expression of IL-1β, whereas IL-1β was elevated in females in lung culture. Females presented increased cell infiltration/activation. In the kidney, males presented increased plasma creatinine, increased expression of caspase-3, and increased leukocyte migration to renal tissue than females.

**Conclusions:**

The authors observed an organ and sex-dependent response to the slow-induction of BD. These results suggest that management strategies should consider the sex of the donor to achieve the best treatment, improving graft quality.

## Highlights

• Slow induction of BD affects male and female organs differently.
• Reduced lung function was observed in females with higher leukocyte infiltration.
• Greater kidney injury was observed in males, with higher plasma creatinine levels.

## Background

Biological sex has been widely shown to impact disease onset and progression, with males being more susceptible to infectious diseases, whereas females present more susceptibility to autoimmune disorders.^1^^,^^2^ These disparities are associated with differences in the innate immune response, which are strongly influenced not only by genetic and epigenetic differences between males and females but also by sex hormones.^3^

In the transplantation field, sex also plays an important role in transplant outcomes. Clinical studies have highlighted how sex-mismatched transplantation is associated with a poor post-transplant prognosis, especially in the lungs.^4^ and kidneys.^5^ The majority of organs allocated for transplantation are from donation after Brain Death (BD). BD is characterized by increased intracranial pressure, leading to herniation of the brain stem, usually as a consequence of trauma or cerebrovascular accidents, resulting in several systemic alterations.^6^

In an experimental model of fast induction of BD simulating cranial trauma, the group has previously shown that, compared with males, females present an increased inflammatory response associated with the loss of sex hormones, affecting especially the heart and the lungs.^7^, ^8^, ^9^, ^10^ Additionally, studies from Rebolledo et al.^11^ and van Zanden et al.^12^ in male animals revealed how the etiology of BD affects thoracic and abdominal organs differently and concluded that kidneys presented increased damage after slow induction of BD, while the reverse occurred to the lungs. In that sense, considering that males and females have divergent responses to the fast induction of BD and that the slow onset affects specific organs in a different manner, the present study aimed to investigate how the slow induction of BD affects males and female rats, with a focus on the lungs and kidneys.

## Methods

### Animals

Female and male Wistar rats (8–12-weeks old) from Envigo (The Netherlands) were maintained at 23±2 °C, with a 12 h light and dark cycle and food and water ad libitum. The animals received care under the Principles of Laboratory Animal Care (NIH Publication n° 86–23, revised 1985) and the Dutch Law on Experimental Animals Care. This study was approved by the Institutional Animal Care and Use Committee of the University of Groningen and follows the ARRIVE guidelines.

The animals were divided into 4 groups:

Female naïve (*n* = 4) = control female animals that did not undergo any surgical procedure;
Female BD (*n* = 8) = female animals that underwent BD induction;
Male naïve (*n* = 4) = control male animals that did not undergo any surgical procedure;
Male BD (*n* = 8) = male animals that underwent BD induction.

### Estrous cycle identification

The female animals were used in the estrous and proestrous phases of the estrous cycle (heat period). The cellular profile was assessed via vaginal lavage with a Pasteur pipette filled with 10 µL of saline solution (NaCl 0.9 %) and stained with 10 µL of crystal violet (5 %). The phase of the cycle was identified via optical microscopy.

### Brain death induction

All animals were anesthetized with a mixture of 5 % isoflurane and maintained with 2 % isoflurane. The temperature was monitored with a rectal probe and maintained at 37 °C via a heating mat. The jugular vein was cannulated for fluid administration and any necessary influx of vasoactive drugs for hemodynamic stabilization. The carotid artery was cannulated for blood sampling and blood pressure measurements. A tracheostomy was performed, and the animals were connected to a small animal ventilator (Harvard Apparatus, model 683; Holliston, MA, USA) at a frequency of 70 breaths/min and a tidal volume of 10 mL/kg. For BD induction, a Fogarty® 4F catheter was inserted intracranially and slowly inflated over a span of 30 min. BD was confirmed by bilateral mydriasis and apnea. After BD confirmation, anesthesia was stopped, and fluid administration was initiated (saline solution, NaCl 0.9 %, 2 mL/h) for the remaining 4h. When necessary, noradrenaline was administered for hemodynamic stabilization. Blood samples were collected at the beginning and at the end of the 4 h period. Nonmanipulated (naïve) animals were used as controls.

Around 5 minutes before the end of the experiment, the animals received an intravenous injection of a muscle relaxer (suxamethoniumchloride; 0.04 mg/100*g* body weight) and heparin (1 mL; 250 U/mL). After 4 h, animals were exsanguinated, and a whole-body flush (maximum pressure of 30 mmHg) was performed with 40 mL of cold saline. Blood, urine and organs were collected.

### Lung tissue culture (explant)

After BD, the lung fragments were collected and incubated in Dulbecco's Modified Eagle's Medium (DMEM) in a humid atmosphere with 5 % CO_2_ at 37 °C for 24h. The supernatants were collected and stored for further analysis.

### Sex hormone determination

Blood samples were collected before and 4 h after BD induction. The quantification of estradiol, progesterone and testosterone was performed using ELISA kits (Cayman Chemical Company, USA) following the manufacturer's instructions.

### Blood gas analysis

Arterial blood samples obtained from the carotid artery before BD induction and 4 h after BD induction were used for gas analysis. Blood gas was measured via an ABL90 FLEX blood gas analyzer (Radiometer, The Netherlands), and pCO_2_, pO_2_ and lactate were recorded.

### Biochemical analysis

LDH and creatinine were measured in the plasma. The levels of creatinine, Na^+^, K^+^ and urea in the urine were measured. Measurements were performed in accordance with the Clinical Laboratory, University Medical Center Groningen, following standard biochemical methods.

### Creatinine clearance

Urinary and plasmatic creatinine levels obtained from biochemical analyses were used to calculate creatinine clearance. An adapted formula from Armstrong-Jr et al., (2023) was used: Creatinineclearance=(urinecreatinineconcentration[mmoL/L]×urineproductionflow[mL/h]/plasmacreatinineconcentration[mmoL/L])/ratweight[g]).

### IL-1β and IL-6 quantification

IL-1β was measured in the plasma, lung homogenate and explants. IL-6 was measured in the plasma, lung explants and kidney homogenate. Quantifications were performed using DuoSet ELISA commercial kits (R&D Systems, USA) in accordance with the manufacturer´s specifications.

### Immunohistochemistry analyses

After 4 h of BD, the lungs and kidneys were collected and subsequently embedded in paraffin. Paraffin sections (4 µm) were prepared for staining. Deparaffinization was performed with xylene and ethanol. For antigen retrieval, the slides were immersed for 3 h at 60 °C in EDTA (1 mM), pH8. The following primary antibodies were used for the lungs: MPO (1:50 ‒ PA1054 – Boster, USA), iNOS (1:100 ‒ AB3523 – Abcam, UK), and eNOS (1:100 ‒ AO1604–2 – Boster, USA). ICAM-1 (1:50 ‒ PB9018 – Boster, USA) and VCAM (1:100 ‒ AO119–2 – Boster, USA). Kidney markers were MPO (1:100, PA1054 – Boster, USA), MMP-9 (1:100, PB9668 – Boster, USA), eNOS (1:100, AO1604–2 – Boster, USA), iNOS (1:100, AB3523 – Abcam, UK) and caspase-3 (1:100, AB4051 – Abcam, UK). The sections were incubated with primary antibodies overnight at 4 °C. The sections were then incubated with a secondary HRP-conjugated antibody (1:200 – Boster ‒ BA1054) at 37 °C for 1h30 to 2 h, and later with a peroxidase substrate. Hematoxylin was used for counterstaining. NIS-Element-BD (Nikon, Japan) software was used for the analyses. MPO, lung iNOS and caspase-3 are expressed as the number of cells per mm^2^. ImageJ software was used for cell quantification. eNOS, ICAM-1 and VCAM-1 are expressed as the stained area per vessel area. Kidney iNOS and MMP-9 are expressed as the stained area per total area.

### Gene expression

RNA was extracted from the kidney and lung using TRIzol reagent (Invitrogen). The yield of extracted RNA was analyzed with a NanoDrop 1000 spectrophotometer (NanoDrop Technologies, USA), and the quality was assessed via RNA electrophoresis. The extracted RNA was reverse transcribed via random hexamer primers (Thermo-Fisher, USA) at 37 °C for 50 min. Real-time quantitative polymerase chain reaction (qPCR) was conducted using specific primers from SYBR Green (Applied Biosystems, The Netherlands) (Table 1) and a Quant Studio 7 Flex qPCR machine (Applied Biosystems, The Netherlands). The cycle configuration was: 1 cycle of 10-min at 95 °C and 40 consecutive cycles of 15 s at 95 °C and 1 min at 60 °C.

**Table 1:** RT-PCR SYBR Green primers.

**n**	**Forward sequence**	**Reverse sequence**
β-actin	5´-GGAAATCGTGCGTGACATTAAA-3´	5´-GCGGCAGTGGCCATCTC-3
eNOS	5´-AGTCCTCACCGCCTTTTCCA-3	5´-GCACGCGGTGAACCTCC-3
IL-6	5´-CCAACTTCCAATGCTCTCCTAATG-3´	5´-TTCAAGTGCTTTCAAGAGTTGGAT-3
Caspase-3	5´-GCATGCCAGAAGATACCAGTGG-3	5´-AGTTTCAGCATGGCGCAAA-3
BCL-2	5´-CTGGGATGCCTTTGTGGAA-3	5´-TCAGAGACAGCCAGGAGAAATCA-3
KIM-1	5´-AGAGAGAGCAGGACACAGGCTTT-3	5´-ACCCGTGGTAGTCCCAAACA-3
IL-1β	5´-CAGCAATGGTCGGGACATAGTT-3	5´-GCATTAGGAATAGTGCAGCCATCT-3

RT-PCR, Real Time Polymerase Chain Reaction; eNOS, Endothelial Nitric Oxide Synthase); IL-6, Interleukin 6; BCL-2, B cell Lymphoma-2; KIM-1, Kidney Injury Marker-1; IL-1β, Interleukin-1 Beta.

### Statistical analysis

The data are expressed as the mean ± Standard Error of the Mean (SEM) or as the median and the maximum and minimum. The data were analyzed with GraphPad Prism Version 10.3.1. For mean arterial pressure, noradrenaline, hormone quantification, gene expression, urinary creatinine, urea, K^+^ and Na^+^ were compared using the Mann-Whitney test. For all other graphs, groups were compared using two-way ANOVA followed by the post hoc-test of the two-stage linear step-up procedure of Benjamin, Krieger and Yekutieli.

## Results

### Hemodynamic parameters

**Fig. 1:**
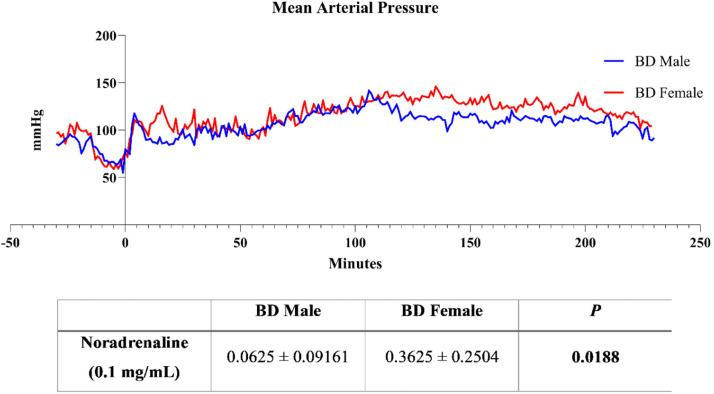
Mean arterial pressure measurements and volume of noradrenaline (0.01 mg/mL) administered to animals during 4 h of BD. BD female, female rats subjected to brain death (*n* = 8); BD male, male rats submitted to brain death (*n* = 8). MAP data are expressed as mean only and noradrenaline values represent the means and Standard Errors of the Means (SEMs).

After balloon catheter insertion, insufflation started once all the animals were stable, with a Mean Arterial Pressure (MAP) of 80 mmHg, and lasted for 30 min (data presented before the Y axis) (Fig. 1). During this period, an increase to 100 mmHg was observed in the first 10 min of induction, followed by a decrease to approximately 50 mmHg and a reestablishment of the MAP to 80 mmHg.

After BD confirmation, the MAP was kept sTable above 80 mmHg by the administration of intravenous noradrenaline, resulting in no difference between males and females. However, the authors observed that females required higher volumes of noradrenaline to reach the desired MAP.

### Hormonal profile

#### Estradiol, progesterone and testosterone

**Fig. 2:**
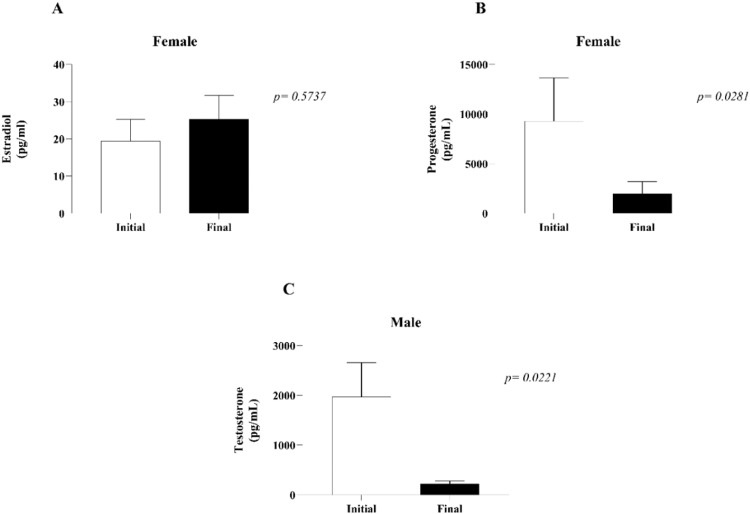
Plasma quantification of estradiol (A) and progesterone (B) in females and testosterone in males (C). Plasma samples were collected before and 4 h after BD induction. The values represent the means and Standard Errors of the Means (SEMs) of 8 animals per group.

In females, there was no change in estradiol plasma levels at the initial (0h) or final (4h) measurements (A), whereas progesterone was significantly lower after 4 h of BD than at the initial values (B). In males, plasma levels of testosterone (C) were also lower at the final time point (4h) in comparison to the initial time point (0h) (Fig. 2).

### Plasma measurements of IL-1β, IL-6, ldh and lactate

To evaluate the systemic inflammatory profile, the plasma levels of IL-1β and IL-6 were quantified. There was a significant increase in the concentrations of both cytokines in males after BD compared to naïve. Compared with naïve females, females also presented higher values of these cytokines after BD. The plasma concentrations of LDH and lactate were also evaluated. No difference was observed in LDH, whereas lactate was increased at 4 h after BD induction compared with the initial values in males and females. In regard to sex difference, females presented higher concentrations of lactate than males did at the final measurement point (Table 2).

**Table 2:** Systemic measurement of IL-1β, IL-6, LDH and lactate. Blood samples were collected at the beginning (0h) and at the end (4h) of the BD period. Naïve, nonmanipulated animals (*n* = 4); BD, rats subjected to Brain Death (*n* = 8).

Plasma					
	Male		Female		
	Naïve	BD	Naïve	BD	P
**IL-1β (pg/mL)**	11.22±0.22	36.04±7.15^a^	11.43±1.37	21.14±3.05	P_BD_ = 0.0063
					P_sex_ = 0.2090
					P_interac_ = 0.1969
**IL-6 (pg/mL)**	9.47±0.00	2060±634.95^a^	9.47±0.00	1521±516.67	P_BD_ = 0.0070
					P_sex_ = 0.6549
					P_interac_ = 0.6546
**LDH (U/L)**	265.33±26.46	568.25±148.70	241.50±68.53	339.62±90.54	P_BD_ = 0.1649
					P_sex_ = 0.3746
					P_interac_ = 0.4698
**Blood**					
	**Male**		**Female**		
	**Initial**	**Final**	**Initial**	**Final**	**P**
**Lactate**	1.462±0.17	2.608±0.55^b^^,^^c^	1.421±0.18	4.400±0.53^b^	P_BD_ < 0.0001
					P_sex_ = 0.0383
					P_interac_ = 0.0306

The values represent the means and standard errors of the means (SEMs).

a *p* < 0.05 compared with the naïve group.

b *p* < 0.05 compared with the initial values.

c *p* < 0.05 compared with females. IL-1β, Interleukin-1β; IL-6, Interleukin-6; LDH, Lactate Dehydrogenase.

### Blood gas analyses

Blood gas measurements before and after BD were evaluated. Both males and females presented a reduction in pO_2_ and pCO_2_ after 4 h of BD. However, females presented even lower levels of pO_2_ in comparison to males, suggesting a worsening of lung function (Table 3).

**Table 3:** Blood gas measurements. Blood samples were collected at the beginning (0h) and at the end (4h) of the BD period. BD female, female rats subjected to brain death (*n* = 8); BD male, male rats submitted to brain death (*n* = 8).

		**Male**	**Female**	**P**
**pO^2^ (mmHg)**	0h	453.31± 24.30	502.07±9.01	P_time_ < 0.0001
	4h	306.96±37.80^a,b^	198.18±26.18^a^	P_sex_ = 0.2653
				P_interac_ = 0.0059
**pCO^2^ (mmHg)**	0h	60.73±6.6	58.92±4.14	P_time_ < 0.0001
	4h	30.60±5.2^a^^,^^b^	26.01±2.41^a^	P_sex_ = 0.5158
				P_interac_ = 0.7773

The values represent the means and standard errors of the means (SEMs).

a *p* < 0.05 compared with the initial values.

b *p* < 0.05 compared with females. pO^2^, Partial pressure of O^2^; pCO^2^, Partial pressure of CO^2^.

### Quantification of interleukins in the lung homogenate and explants

**Fig. 3:**
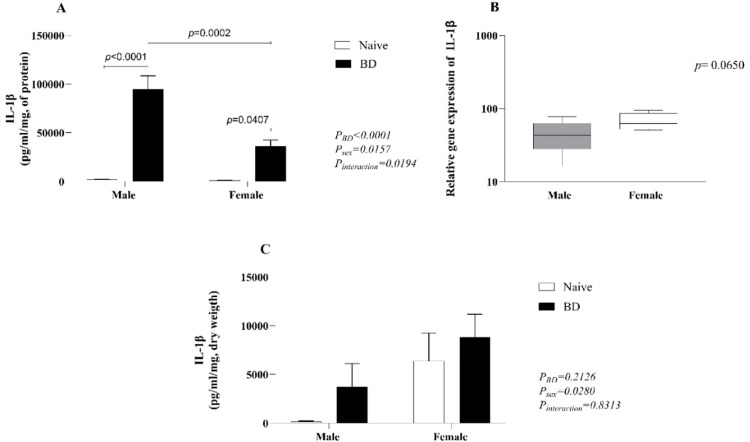
Quantification of IL-1β in lung homogenates (A) and explants (C) and gene expression (B). Naïve, nonmanipulated animals (*n* = 4); BD, rats subjected to brain death (*n* = 8). The values represent the means and Standard Errors of the Means (SEMs) (A and C) and median and the maximum and minimum (B). IL-1β, Interleukin-1β.

**Fig. 4:**
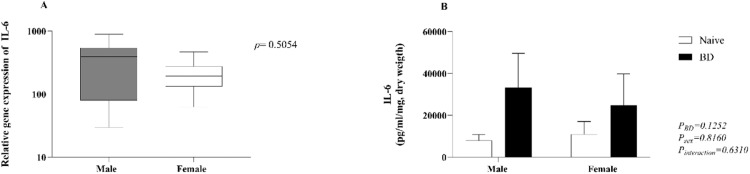
Gene expression (A) and concentration of IL-6 (B) in lung explants. Naïve, nonmanipulated animals (*n* = 4); BD, rats subjected to brain death (*n* = 8). The values represent the means and Standard Errors of the Means (SEMs) (B) and median and the maximum and minimum (A). IL-6, Interleukin-6*.*

In the lung homogenate (A), there was an increase in IL-1β in both males and females compared with that in naïve animals, whereas males presented significantly higher values than females after BD. According to the results of the PCR analyses (B) and explants (C), females presented slightly higher concentrations and increased gene expression of IL-1β than males (Fig. 3). No significant difference in the level of IL-6 was observed among the groups (Fig. 4).

### Leukocyte infiltration

#### MPO

**Fig. 5:**
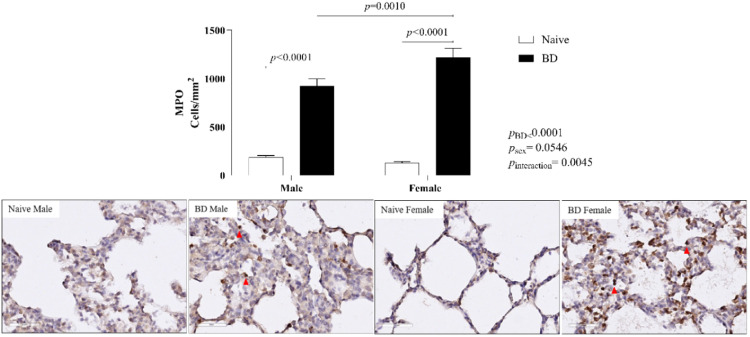
Protein expression of MPO in lung tissue (red arrow). Naïve, nonmanipulated animals (*n* = 4); BD, rats subjected to brain death (*n* = 8). The values represent the means and Standard Errors of the Means (SEMs). MPO, Myeloperoxidase. Representative photomicrographs (×40) of each group.

To evaluate leukocyte infiltration into lung tissue, the protein expression of Myeloperoxidase (MPO) was assessed. There was an increase in the number of infiltrated cells in both the male and female BD groups compared with the naïve groups. Concerning sex difference, females presented even higher values than males did after BD (Fig. 5).

#### Adhesion molecules

**Fig. 6:**
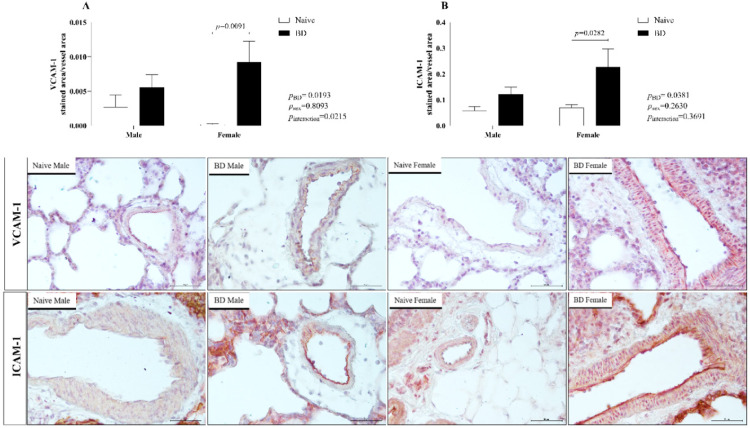
Protein expression of VCAM-1 and ICAM-1 in lung tissue. Naïve, nonmanipulated animals (*n* = 4); BD, rats subjected to brain death (*n* = 8). The values represent the means and Standard Errors of the Means (SEMs). Representative photomicrographs (×20) of each group. VCAM-1, Vascular Cell Adhesion Molecule; ICAM-1, Intercellular Adhesion Molecule.

Additionally, the protein expression of adhesion molecules was evaluated. Both VCAM-1 and ICAM-1 were more highly expressed in BD female animals than in the respective naïve animals, which did not occur in male rats. No difference was observed between the sexes (Fig. 6).

### Expression of nitric oxide synthases in lung tissue

#### iNOS

**Fig. 7:**
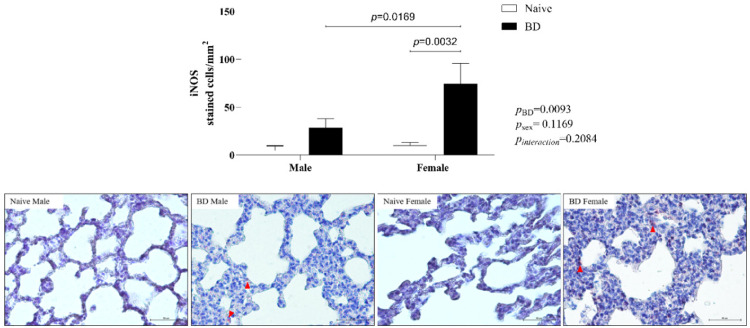
Protein expression of iNOS in lung tissue (red arrow). Naïve, nonmanipulated animals (*n* = 4); BD, rats subjected to brain death (*n* = 8). The values represent the means and Standard Errors of the Means (SEMs). Representative photomicrographs (×20) of each group. iNOS, Inducible Nitric Oxide Synthase.

Inducible Nitric Oxide Synthase (iNOS) was also evaluated in the lung. There was an increase in the number of stained cells in the BD female group compared with those in the female naïve and BD male groups (Fig. 7).

#### eNOS

**Fig. 8:**
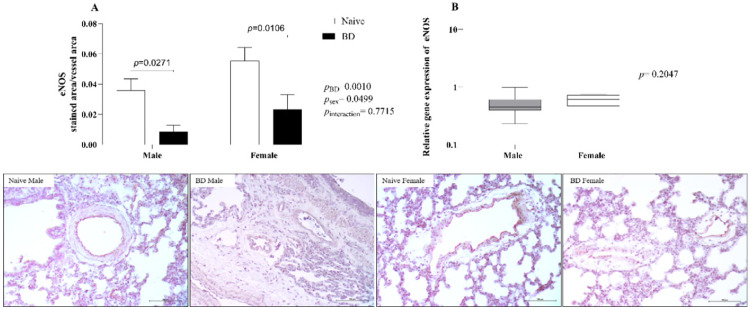
Protein expression of eNOS in lung tissue (A) and gene expression (B). Naïve, nonmanipulated animals (*n* = 4); BD, rats subjected to brain death (*n* = 8). The values represent the means and Standard Errors of the Means (SEMs) (A) and median and the maximum and minimum (B). Representative photomicrographs (×40) of each group. eNOS, Endothelial Nitric Oxide Synthase).

With respect to the protein expression of endothelial Nitric Oxide Synthase (eNOS) in lung tissue (A), both males and females presented reduced expression after BD in relation to the respective naïve animals. Relative gene expression was also evaluated (B), and no difference was observed between the groups (Fig. 8).

### Kidney function

In order to evaluate kidney function, plasma and urinary concentrations of creatinine were quantified. Urinary concentrations of urea, Na^+^and K^+^ were also analyzed (Table 4). In the plasma, males presented higher concentrations of creatinine after BD in comparison to male naïve and BD female animals. The same could be observed in the urine, with males presenting increased values in comparison to females. With respect to the other parameters, K^+^was higher in the males than in the females and no differences were observed in relation to urea and Na^+^.

**Table 4:** Plasmatic values of creatinine. Urinary values of creatinine, urea, Na^+^ and K^+^. Naïve, nonmanipulated animals (*n* = 4); BD, rats subjected to brain death (*n* = 8).

**Plasma**							
	**Male**			**Female**			
	**Naïve**		**BD**	**Naïve**		**BD**	**P**
**Creatinine (umoL/L)**	18.66±0.88		62.50±14.40^a^^,^^b^	18.75±1.10		32.12±3.64	P_BD_=0.0239
							P_sex_=0.2092
							P_interac_=0.2068

**Urine**							
		**Male**			**Female**		**P**
**Creatinine (mmoL/L)**		4.16±0.74			2.16±0.42		*P* = 0.0247
**Urea (mmoL/L)**		183.9 ± 25.3			151.8 ± 19.07		*P* = 0.3282
**Na^+^(mmoL/L)**		25.88±3.71			26.88±4.34		*P* = 0.9608
**K^+^(mmoL/L)**		75.81±10.83			41.56±9.30		*P* = 0.0499

The values represent the means and standard errors of the means (SEMs).

a *p* < 0.05 compared with the naïve group.

b *p* < 0.05 compared with females. Na^+^, Sodium; K^+^, Potassium.

### Quantification of IL-6 in kidney homogenate

**Fig. 9:**
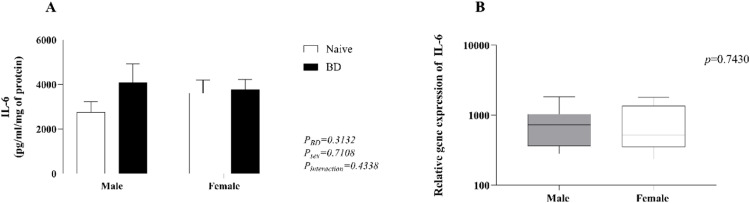
Quantification of IL-6 in kidney homogenates (A) and gene expression (B). Naïve, nonmanipulated animals (*n* = 4); BD, rats subjected to brain death (*n* = 8). The values represent the means and Standard Errors of the Means (SEMs) and median and the maximum and minimum (B). IL-6, Interleukin-6.

IL-6 was quantified in kidney homogenates. No difference was observed between the naïve and BD groups in either sex (A). There were no differences in the gene expression of IL-6 (B) between males and females (Fig. 9).

### Protein expression of MPO and MMP9 in kidney tissue

**Fig. 10:**
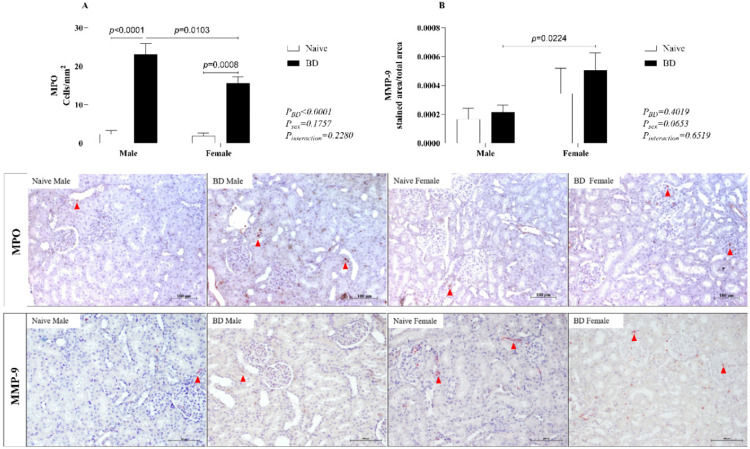
Protein expression of MPO and MMP-9 in kidney tissue (red arrow). Naïve, nonmanipulated animals (*n* = 4); BD, rats subjected to brain death (*n* = 8). The values represent the means and Standard Errors of the Means (SEMs). Representative photomicrographs (×40) of each group. MPO, Myeloperoxidase; MMP-9, Metalloproteinase-9.

In the Myeloperoxidase (MPO) Analysis (A), there was an increase in the number of migrated cells in both males and females after BD compared with that in naïve individuals. In terms of sex difference, males presented greater leukocyte infiltration than females. Metalloproteinase-9 (MMP-9) was also evaluated (B), and there was increased protein expression in BD females compared with BD males (Fig. 10).

### Expression of nitric oxide synthases in kidney tissue

#### iNOS

**Fig. 11:**
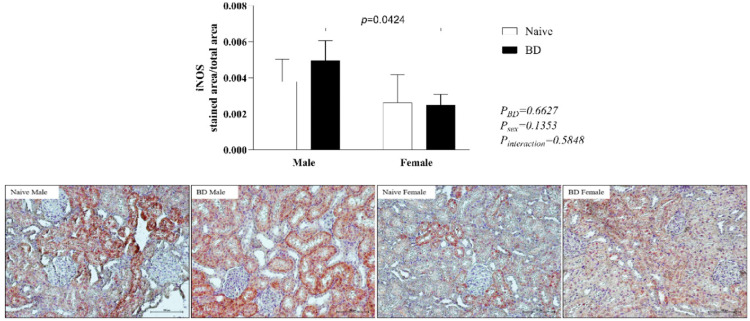
Protein expression of iNOS in kidney tissue. Naïve, nonmanipulated animals (*n* = 4); BD, rats subjected to brain death (*n* = 8). The values represent the means and Standard Errors of the Means (SEMs). Representative photomicrographs (×40) of each group. iNOS, Inducible Nitric Oxide Synthase.

To further analyze renal inflammation, the protein expression of iNOS was analyzed. Compared with females, males presented greater expression of iNOS after BD (Fig. 11).

### Apoptosis markers

**Fig. 12:**
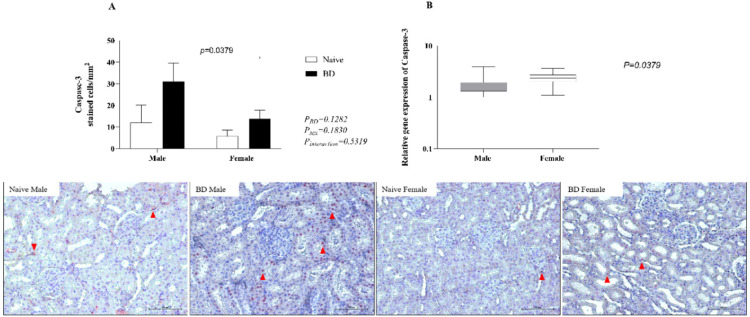
Protein (A) (red arrow) and gene (B) expression of caspase-3 in kidney tissue. Naïve, nonmanipulated animals (*n* = 4); BD, rats subjected to brain death (*n* = 8). The values represent the means and Standard Errors of the Means (SEMs) (A) and median and the maximum and minimum (B). Representative photomicrographs (×40) of each group.

Finally, to analyze kidney apoptosis, the protein and gene expression of caspase-3 was evaluated. After BD, males presented a greater number of stained cells than females (A). However, in gene expression females presented higher expression than males (B) (Fig. 12).

## Discussion

Previous investigations from the group in the fast induction model of BD have shown greater impairment of microcirculation in males, marked by increased platelet aggregation, and a more relevant inflammatory response in females with pronounced cell infiltration to the organs.^7^^,^^8^^,^^13^ The present study highlights how the slow induction of BD may affect males and females in different manners and how BD-induced sex dimorphism can be organ-dependent. The present results indicate increased pulmonary injury in females, with more prominent cell infiltration and reduced blood pO_2_. In the kidneys, however, males presented greater leukocyte infiltration and cell apoptosis, accompanied by higher plasma levels of creatinine.

Several studies have used experimental models of BD,^14^, ^15^, ^16^, ^17^ which primarily consist of the insertion and insufflation of a balloon catheter inside the skull, generating an increase in intracranial pressure and herniation of the brain stem followed by a hypotensive phase. Two distinct BD models are described in the literature: the fast and the slow induction. Fast induction is used to simulate acute trauma to the brain, with rapid expansion of the balloon. In this model, an increase in arterial pressure is observed within the first 60 seconds. The slow induction mimics a hemorrhagic stroke via gradual insufflation of the balloon, resulting in a slow increase in arterial pressure and maintaining some hemodynamic stability.^18^ Studies from Rebolledo et al*.* (2016) and van Zanden et al*.* (2020) compared the impact of slow and fast induction of BD in male rats. Both studies revealed a rapid increase in the Mean Arterial Pressure (MAP) in the fast model, which was not observed in the slow-onset model. Additionally, animals subjected to fast insufflation of the catheter required more inotropic support.

In fact, the group has previously shown that the fast induction of BD leads to an acute increase in the MAP, usually in the first minute, followed rapidly by a hypotensive phase.^13^^,^^19^^,^^20^ In studies directly comparing males and females in the fast model, without hemodynamic control, no differences in hemodynamic behavior were observed between the sexes.^8^ In the present study, the authors used a slow induction model in which the intracranial balloon was gradually insufflated for 30 minutes. Unlike fast induction, the model does not show an acute increase in MAP, and the hypotensive phase can be observed in the last 10 minutes before BD confirmation. This pattern was similar in both groups subjected to BD, regardless of sex. After BD induction, noradrenaline was administered when the MAP reached less than 80 mmHg, and both males and females presented similar MAP patterns during the 4 h of BD. However, female animals require higher levels of noradrenaline to achieve normotensive values, suggesting that after slow induction, females are more hemodynamically unsTable than males.

Another relevant point in the pathophysiology of BD is the loss of the hypothalamus-hypophysis axis, which compromises the donor endocrine system. Clinical studies have highlighted the reduction in T3, T4, insulin and glucocorticoids, and experimental studies have demonstrated a reduction in female sex hormones.^7^^,^^21^ In the literature, little is known about sex hormones in brain-dead males. However, Amado et al.^22^ reported a reduction in testosterone in brain-dead male patients. The present results indicate an acute reduction in testosterone in males after 4 hours of BD compared with the initial values. In females, the authors previously showed that both progesterone and estradiol were reduced 3 hours after fast induction of BD.^7^ In the present study, the authors also observed a significant reduction in progesterone in the females; however, the estradiol concentrations after 4 hours remained similar to the initial values. This behavior in the concentration of estradiol could be due to greater stress response of the females to the slow induction of BD, as the 30 min between catheter insertion and BD confirmation could represent a window in which the hormone is acutely released into the bloodstream. In this context, before the surgical procedure, females were selected for the phase of the estrous cycle that presents peak levels of estradiol. Indeed, studies have shown that females present a greater stress response during phases of the estrous cycle with high estradiol concentrations.^23^ Moreover, differences in estradiol and progesterone concentrations may also be related to the metabolism of both hormones. Progesterone is strongly susceptible to enzymatic reduction and is rapidly transformed into subproducts that take part in the synthesis of other steroid hormones,^24^ whereas estradiol is converted from testosterone and estrone much later in the pathway.^25^

Consistently, several studies have highlighted the increase in inflammatory mediators after BD and its association with a poor prognosis after transplantation.^26^^,^^27^ More recently, Belhaj et al.^28^, in a porcine model, reported that increased serum levels of IL-1β and IL-6 after BD were associated with increased renal injury. The present results revealed a similar scenario, with increased plasma concentrations of IL-1β and IL-6 after BD groups compared with the respective controls. Furthermore, compared with the initial measurements, both males and females presented reduced pO_2_ and pCO_2_ at 4 hours, with a more relevant decrease in both gases in the females. Ricardo-da-Silva et al. (2024), in the same experimental model, also reported reduced pO_2_ in females after BD; however, that difference was not maintained during *ex vivo* lung perfusion. With respect to LDH, no difference was observed, but females presented higher levels of lactate at the final measurement point compared to males. The authors suggest that in females, increased lactate could be a result of noradrenaline-derived vasoconstriction and reduced pO_2_, leading to hypoxia in peripheral tissue. Lactate is a known byproduct of glycolysis in an anaerobic environment due to insufficient oxygen delivery, .^29^^,^^30^ and compared with other catecholamines, norepinephrine administration is associated with increased serum lactate levels.^31^ Moreover, lactate may also result from epinephrine-induced aerobic glycolysis via stimulation of Na^+^, K^+^-ATPase activity.^32^

The systemic imbalance triggered by BD also locally compromises graft function. In the lungs, changes in vascular resistance and MAP lead to neurogenic pulmonary edema and inflammation.^33^ Studies from Breithaupt-Faloppa et al*.* (2016) and Simão et al*.* (2016) have shown that, after fast-induced BD, females present more exacerbated pulmonary inflammation than males do, which is associated with a rapid decrease in estradiol. Estradiol is an important regulator of the female immune response and is related to an increased cell-mediated immune response.^34^^,^^35^ and reduced release of proinflammatory mediators, such as IL-6, IL-1β and TNF-α.^36^ Indeed, these results showed an increased neutrophil infiltration into the lungs of female BD animals compared to males, as shown by higher MPO expression, after BD. The authors also quantified macrophage activation by counting iNOS-marked cells. Similar to neutrophils, females presented an increased number of activated cells compared with female controls and BD males. Leukocyte infiltration was accompanied by increased expression of adhesion molecules ICAM-1 and VCAM-1 in females. These findings suggest that females present a more exacerbated cellular immune response in the lungs after BD, independent of induction time. In lung homogenate, the authors observed a lower concentration of IL-1β in females, which could be associated with the maintenance of estradiol levels after BD. Indeed, estradiol has been shown to modulate neutrophil recruitment to the site of ongoing inflammation,^37^ but also promote its anti-inflammatory activity.^34^ In lung explant, however, IL-1β quantification revealed higher values in females than in males after 24h. Moreover, previous studies in the same model comparing males and females, followed by 4 h of *ex vivo* lung perfusion, have shown increased levels of IL-1β in the perfusate of female's lungs.^38^ Such scenarios could be a result of the lack of estradiol in the *ex vivo* system or culture medium, triggering a proinflammatory response in the infiltrated immune cells. Indeed, low levels of estradiol have been previously associated with increased release of IL-1β.^39^ Additionally, several clinical studies have highlighted the worst patient and graft survival after donation between female donors and male recipients, whereas sex-matched transplant recipients presented superior outcomes.^40^^,^^41^ These clinical results reiterate the findings that female lungs presented inferior quality, highlighted by decreased pO_2_ and increased leukocyte infiltrate, and that these grafts may perform worse in environments with low or non-existing estradiol.

Lastly, endothelial NOS expression was also analyzed in lung tissue, and both sexes presented lower eNOS levels after BD induction than their respective naïve groups. These results corroborated previous findings showing that, in the lung, both males and females presented reduced expression of eNOS after fast induction of BD.^10^^,^^42^

With respect to renal damage after slow induction of BD, the present results indicate a worsening of kidney function in males, marked by increased plasma and urinary creatinine levels, cell apoptosis and increased infiltration of immune cells. It has been previously shown that inflammation is closely linked to mechanisms that lead to coagulation derangement through thrombin and fibrin formation, especially those related to tissue factor release.^43^ IL-6 has also been shown to play a role in the initiation of the coagulation pathway.^44^ Coagulopathy is a known consequence of BD and is especially characterized by temporary hypercoagulation.^45^ In an experimental model, Correia et al. (2020) described sex differences in the coagulation process after BD, with males presenting greater platelet aggregation, increased clot firmness and reduced microvascular perfusion than females. Indeed, in the same model of slow induction of BD, Armstrong Jr et al. (2023) reported increased expression of eNOS in females compared with males, suggesting better maintenance of flow in females. eNOS is expressed in the endothelium and promotes vasodilation, and estradiol is associated with increased expression and activity of Enos.^46^^,^^47^ In the kidney, microcirculation is a key point in the development of Acute Kidney Injury (AKI), and hypoperfusion due to microthrombi formation could lead to disruption of cellular homeostasis, culminating in cell death.^48^ In the present study, BD males presented increased protein expression of the apoptosis marker caspase-3, whereas gene expression was greater in females. Armstrong Jr et al. (2023) also reported a pro-apoptotic state in male kidneys before machine perfusion. Caspase-3 expression has been linked to the early onset of AKI,^49^ and these results suggest that such injury may occur earlier in males and later in females. In this context, clinical studies have highlighted a greater risk of AKI in male BD donors before transplantation.^50^ Apoptosis is also known to potentiate inflammation, especially by promoting leukocyte recruitment through the release of chemokines in the form of apoptotic extracellular vesicles.^51^ Indeed, the present results revealed increased leukocyte infiltration in both sexes after BD, with more pronounced neutrophil and macrophage mobilization in males, as indicated by increased expression of MPO and iNOS. Finally, regarding MMP-9, no difference was observed between the control and BD groups in either sex, whereas BD females presented increased protein expression compared with BD males. These results suggest that increased expression of MMP-9 is related to female sex and not to BD induction. Studies have shown that the activation of estradiol receptors is related to increased MMP-9 expression.^52^

### Perspective and significance

Finally, the present study provides an understanding of how the slow induction of BD affects males and females rats in a divergent manner. Unlike fast induction, the model does not show a reduction in estradiol in females, modifying the female response to the systemic and local imbalance triggered by BD. More importantly, these results have shown that tissue damage is sex dependent, and that each organ is differently affected in male and female animals. These insights provide an experimental perspective on how donor treatment and management strategies should be organ-focused and consider the cause of BD, as well as the sex of the donor.

## Ethics approval and consent to participate

The animal study was reviewed and approved by the Institutional Animal Care and Use Committee of the University of Groningen (IACUC-RUG).

## Consent for publication

Not applicable.

## Availability of data and materials

The datasets used and/or analyzed during the current study are available from the corresponding author upon reasonable request.

## Authors’ contribution

MVS: Participated in research design, animal operations, laboratory analyses, data analyses and writing the manuscript. RAJ: Participated in laboratory analysis, data analyses and revision of the manuscript. MvZ, FYRS, LFA, MMAR, CJC: Participated in analyzing data and revising the manuscript. PO: Participated in performing animal operations and laboratory analyses. LFPM: Participated in research design, supervised the study and revision of the manuscript. HL, ACBF: Participated in research design and supervised the study and writing of the manuscript.

## Funding

The author(s) declare financial support was received for the research, authorship, and/or publication of this article. LFPM is a fellow researcher of the CNPq. The author MVS is a scholarship holder by Fundação de Amparo à Pesquisa do Estado de São Paulo ‒ FAPESP ‒ 2020/11,211–6 and later 2021/07,455–0. The author FYRS is a scholarship holder by FAPESP – 2021/13,020–6. The University of Groningen's Graduate School of Medical Sciences also provided funding for this project.

## Conflicts of interest

The authors declare that the resessarch was conducted in the absence of any commercial or financial relationships that could be construed as a potential conflict of interest.

## Appendix Supplementary materials

Image, application 1
