# Supplementary material for: Sex differences in kidney and lung status in an animal model of brain death
Source: Clinics (Sao Paulo). 2025 Mar 26;80:100623. doi: 10.1016/j.clinsp.2025.100623 (PMC11985142; doi:10.1016/j.clinsp.2025.100623)
Supplement: Supplementary file 1 [file mmc1.docx]

**CLINICS-D-25-00069_Complementary data**

**Results**

Although males present slightly lower values of creatinine clearance than females, no significant difference was observed.

**Complementary figure 1** – Creatinine clearance of males (n=8) and females (n=8). The values represent the means and standard errors of the means (SEMs).
